# Incidence of Respiratory Syncytial Virus–Associated Lower Respiratory Tract Illness in Infants in Low- and Middle-Income Regions During the Coronavirus Disease 2019 Pandemic

**DOI:** 10.1093/ofid/ofad553

**Published:** 2023-12-01

**Authors:** Samantha Fry, Kulkanya Chokephaibulkit, Sridevi Pallem, Ouzama Henry, Yongjia Pu, Agnes Akawung, Joon Hyung Kim, Emad Yanni, Antonella Nadia Tullio, Linda Aurpibul, Christine Mui Fong Lee, Ana Ceballos, Khalequ Zaman, Ivonne Abadía de Regalado, Khatija Ahmed, Diana Andrea Arias Fernandez, Sri Wahyu Taher, Juliana Caccavo, Conrado Milani Coutinho, Ulises D’Andrea Nores, Tirza De León, Emily Christine D’Silva, Mara De Bernardi, Pablo Dieser, Andrea Falaschi, Clara del Carmen Flores Acosta, Angela Gentile, Ik Hui Teo, Sheena Kotze, Eduardo López-Medina, Ruben Luca, Maria Florencia Lucion, Jacinto Blas III V Mantaring, Bladimir Marín, Malahleha Moelo, Marisa Márcia Mussi-Pinhata, Jorge Pinto, Thanyawee Puthanakit, Osvaldo Reyes, Maria Fernanda Roa, María Teresa Rodriguez Brieschke, Camilo Enrique Rodriguez, Juan Nicolas Rodriguez Niño, Alexandre Vargas Schwarzbold, Alexandra Sierra Garcia, Lavitha Sivapatham, Ruey Soon, Juan Carlos Tinoco, Jesús Arnulfo Velásquez Penagos, Gaël Dos Santos

**Affiliations:** Department of Paediatrics and Child Health, Family Centre for Research with Ubuntu, Stellenbosch University, Cape Town, South Africa; Department of Pediatrics, Faculty of Medicine Siriraj Hospital, Mahidol University, Bangkok, Thailand; Keyrus Life Science c/o GSK, Wavre, Belgium; GSK, Rockville, Maryland, USA; GSK, Rockville, Maryland, USA; Keyrus Life Science c/o GSK, Wavre, Belgium; GSK, Rockville, Maryland, USA; GSK, Rockville, Maryland, USA; GSK, Rockville, Maryland, USA; Research Institute for Health Science, Chiang Mai University, Chiang Mai, Thailand; Department of Obstetrics and Gynaecology, Sarawak General Hospital, Kuching, Malaysia; Instituto Médico Río Cuarto, Río Cuarto, Córdoba, Argentina; International Centre for Diarrhoeal Disease Research (icddr, b), Dhaka, Bangladesh; Policentro de Salud de Juan Diaz, Juan Diaz, Panama; Setshaba Research Centre, Soshanguve, South Africa; Faculty of Health Sciences, Department of Medical Microbiology, University of Pretoria, Pretoria, South Africa; Hospital San José, Bogotá, Colombia; Simpang Kuala Health Clinic, Alor Setar, Malaysia; Donación Francisco Santojanni Hospital, Buenos Aires, Argentina; Department of Gynecology and Obstetrics, Hospital das Clínicas da Faculdade de Medicina de Ribeirão Preto, Universidade de São Paulo, Ribeirão Preto, Brazil; Instituto Médico Río Cuarto, Río Cuarto, Córdoba, Argentina; Maternity Hospital José Domingo De Obaldia, San Pablo Viejo, Panama; Department of Obstetrics and Gynecology, Ampang Hospital, Selangor, Malaysia; Donación Francisco Santojanni Hospital, Buenos Aires, Argentina; Instituto Médico Río Cuarto, Río Cuarto, Córdoba, Argentina; Dr Ramon Carrillo Hospital, Mendoza, Argentina; Dr Diego Paroissien Hospital, Mendoza, Argentina; Dr José E. González University Hospital, Autonomous University of Nuevo León, Monterrey, Mexico; Epidemiology Department, Hospital de Niños Dr Ricardo Gutiérrez, Buenos Aires, Argentina; Hospital Ampang, Kuala Lumpur, Malaysia; Synexus Stanza Clinical Research Centre, Pretoria, South Africa; Centro de Estudios en Infectología Pediátrica, Department of Pediatrics, Universidad del Valle, Valle del Cauca, Colombia; Clinica Imbanaco, Grupo Quironsalud, Cali, Colombia; Hospital F. F. Santojanni C1407, Buenos Aires, Argentina; Epidemiology Department, Hospital de Niños Dr Ricardo Gutiérrez, Buenos Aires, Argentina; Department of Clinical Epidemiology, University of the Philippines, Philippine General Hospital, Manila, Philippines; Hospital San José, Bogotá, Colombia; Setshaba Research Centre, Soshanguve, South Africa; Department of Pediatrics, Ribeirão Preto Medical School, University of São Paulo, Ribeirão Preto, Brazil; Department of Pediatrics, Federal University of Minas Gerais, Belo Horizonte, Brazil; Department of Pediatrics and Center of Excellence for Pediatric Infectious Diseases and Vaccines, Faculty of Medicine, Chulalongkorn University, Bangkok, Thailand; Santo Tomás Hospital, Panama City, Panama; Centro de Vacunación Internacional S.A., La Chorrera, Panama; Member of the Sistema Nacional de Investigadores (SNI), Panama City, Panama; Department of Pediatrics, University Hospital Fundación Santa Fe de Bogotá, Bogotá, Colombia; Donación Francisco Santojanni Hospital, Buenos Aires, Argentina; Department of Gynecology and Obstetrics, University Hospital Fundación Santa Fe de Bogotá, Bogotá, Colombia; School of Medicine, University of the Andes, Bogotá, Colombia; Department of Gynecology and Obstetrics, University Hospital Fundación Santa Fe de Bogotá, Bogotá, Colombia; Hospital Universitário de Santa Maria, Centro de Pesquisa Clínica, Universidade Federal de Santa Maria, Santa Maria, Brazil; Centro de Estudios en Infectología Pediátrica, Department of Pediatrics, Universidad del Valle, Valle del Cauca, Colombia; Clinica Imbanaco, Grupo Quironsalud, Cali, Colombia; Department of Obstetrics and Gynecology, Ampang Hospital, Ampang, Malaysia; Department of Obstetrics and Gynecology, Sabah Women's and Children's Hospital, Kota Kinabalu, Malaysia; General Hospital of Durango, Durango, Mexico; San Vicente Fundación University Hospital, Medellin, Colombia; GSK, Wavre, Belgium

**Keywords:** epidemiology, incidence, infants, lower respiratory tract illness, respiratory syncytial virus

## Abstract

**Background:**

Incidence data of respiratory syncytial virus–associated lower respiratory tract illness (RSV-LRTI) are sparse in low- and middle-income countries (LMICs). We estimated RSV-LRTI incidence rates (IRs) in infants in LMICs using World Health Organization case definitions.

**Methods:**

This prospective cohort study, conducted in 10 LMICs from May 2019 to October 2021 (largely overlapping with the coronavirus disease 2019 [COVID-19] pandemic), followed infants born to women with low-risk pregnancies for 1 year from birth using active and passive surveillance to detect potential LRTIs, and quantitative reverse-transcription polymerase chain reaction on nasal swabs to detect RSV.

**Results:**

Among 2094 infants, 32 (1.5%) experienced an RSV-LRTI (8 during their first 6 months of life, 24 thereafter). Seventeen (0.8%) infants had severe RSV-LRTI and 168 (8.0%) had all-cause LRTI. IRs (95% confidence intervals [CIs]) of first RSV-LRTI episode were 1.0 (.3–2.3), 0.8 (.3–1.5), and 1.6 (1.1–2.2) per 100 person-years for infants aged 0–2, 0–5, and 0–11 months, respectively. IRs (95% CIs) of the first all-cause LRTI episode were 10.7 (8.1–14.0), 11.7 (9.6–14.0), and 8.7 (7.5–10.2) per 100 person-years, respectively. IRs varied by country (RSV-LRTI: 0.0–8.3, all-cause LRTI: 0.0–49.6 per 100 person-years for 0- to 11-month-olds).

**Conclusions:**

RSV-LRTI IRs in infants in this study were relatively low, likely due to reduced viral circulation caused by COVID-19–related nonpharmaceutical interventions.

**Clinical Trials Registration:**

NCT03614676.

## Teaser

In this prospective cohort study, conducted in 10 low- and middle-income countries in 2019–2021, the incidence of respiratory syncytial virus–associated lower respiratory tract illness in infants was low due to reduced viral circulation caused by COVID-19–related nonpharmaceutical interventions.

Lower respiratory tract illnesses (LRTIs) are a major cause of morbidity and mortality in children <5 years old, with respiratory syncytial virus (RSV) being a leading etiology [1, 2]. In regions with temperate climates, RSV causes yearly epidemics during autumn and winter months. In the tropics, RSV circulation is often highest during rainy seasons, with infections occurring throughout the year [3, 4]. RSV caused an estimated 33.0 million acute LRTIs in <5-year-olds in 2019 worldwide, one-fifth of which occurred in the first 6 months of life and >95% in low- and middle-income countries (LMICs) [5]. This resulted in an estimated 3.6 million hospital admissions, 26 300 in-hospital deaths, and 101 400 RSV-associated deaths in <5-year-olds (with almost half in <6-month-olds) [5].

During the first year of the coronavirus disease 2019 (COVID-19) pandemic, the number of infections with RSV and other seasonal respiratory viruses decreased dramatically, due to nonpharmaceutical interventions (NPIs) implemented to reduce the spread of COVID-19, such as lockdowns, physical distancing, reduced social mixing, mask mandates, and increased hand hygiene [6–13]. As these measures relaxed, off-season RSV outbreaks were seen [8, 13–17].

Country-specific estimates of the incidence of laboratory-confirmed RSV-LRTI and RSV-associated hospitalization in infants are scarce or outdated, especially in LMICs [18–20]. Accurate estimates of the RSV-LRTI burden would help plan efficacy trials of prophylactic interventions (eg, RSV vaccine candidates and monoclonal antibodies). They might also support policy decisions on implementing such interventions in LMICs [18–20].

Currently available RSV-LRTI incidence rates (IRs) for LMICs vary widely across studies [5, 21], partly due to differences in diagnostic methods, case definitions, and whether active or passive surveillance was used [20]. Standardization of surveillance methods and case definitions would support comparison of data across different countries. We performed a prospective, multicountry study in LMICs using reliable surveillance mechanisms and standardized case definitions for RSV-associated illnesses to better understand and quantify the RSV burden in infants in LMICs and help plan future RSV vaccine efficacy trials. We estimated the incidence of all, severe, and very severe RSV-LRTI, RSV hospitalization, and all-cause LRTI in infants using World Health Organization (WHO) case definitions [20]. We also aimed to describe coinfections with other respiratory viruses and cord blood RSV-A and RSV-B neutralizing antibody levels among infants developing RSV-LRTI.

The study's primary objectives (frequency of pregnancy outcomes and pregnancy-related and neonatal events of interest) will be reported separately.

## METHODS

### Study Design and Participants

We conducted a prospective cohort study in 10 LMICs (Argentina, Bangladesh, Brazil, Colombia, Malaysia, Mexico, Panama, the Philippines, South Africa, and Thailand) from 30 May 2019 until 20 October 2021, which largely overlapped with the COVID-19 pandemic. We enrolled healthy pregnant women 18–45 years old, with uncomplicated pregnancies, and their live-born infants. Detailed inclusion and exclusion criteria are provided in the Supplementary Materials.

**Figure 1.**
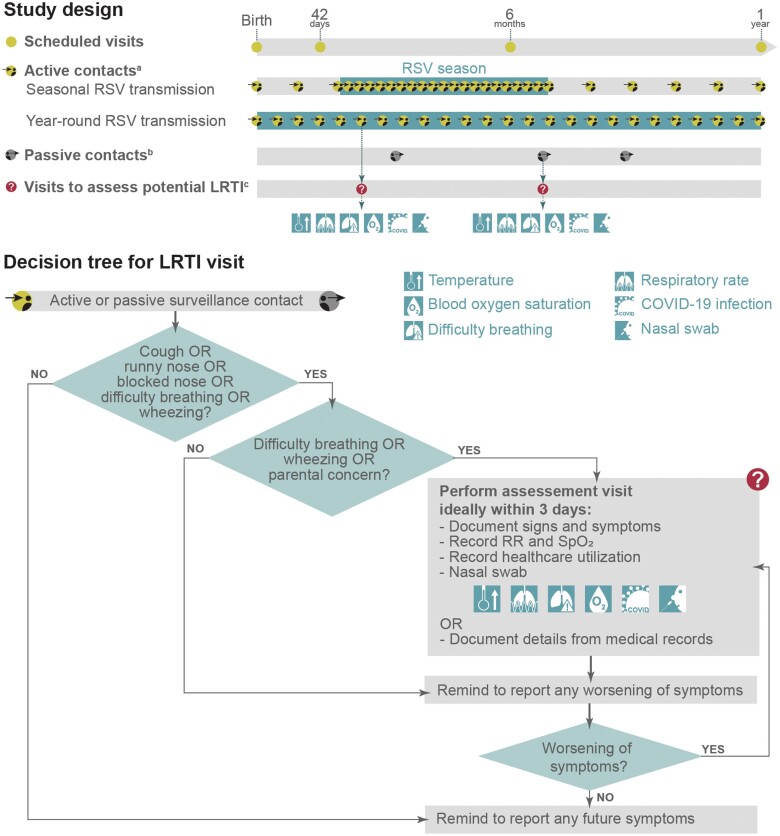
Study design and lower respiratory tract illness (LRTI) surveillance decision tree. ^a^For active contacts, site staff contacted the parents approximately every week during the respiratory syncytial virus (RSV) season and every month during interseason periods in countries with seasonal RSV transmission, and approximately every 2 weeks in countries with year-round RSV transmission. RSV seasonality by country is shown in Figure 2, and information on how seasons were determined is included in the Supplementary Materials. ^b^For passive contacts (which occurred throughout the year independently of RSV seasonality), parents contacted the site staff whenever the infant developed (new) symptoms of a respiratory tract illness (RTI), difficulty breathing, or wheezing; if the infant's symptoms worsened; or if there was parental concern (ie, if the parent[s], legally acceptable representative[s], or designate[s] were concerned about the infant's RTI or general health in the context of the RTI and intended to seek medical care). For both active and passive contacts, a protocol-guided phone script was used to ensure all required information was collected. ^c^The decision for scheduling a visit to assess a possible LRTI and procedures during the visit are explained in the “decision tree for LRTI visit.” Assessment visits were conducted by qualified site staff (ie, physicians, nurses, nurse practitioners, physician's assistants) with documented medical training (ie, medical or nursing license). Abbreviations: COVID-19, coronavirus disease 2019; LRTI, lower respiratory tract illness; RR, respiratory rate; RSV, respiratory syncytial virus; SpO_2_, blood oxygen saturation measured by pulse oximetry in room air, if feasible.

Infants were followed up for 1 year with scheduled visits at birth, 42 days, 6 months, and 12 months of age, contacts for active and passive LRTI surveillance, and ad hoc visits to assess potential LRTIs (Figure 1). No interventional products were administered.

### Patient Consent Statement

All participating women gave written or witnessed/thumbprinted informed consent before participation. Informed consent for infant participation was given by the mother (and father, if required by local regulations) or legally acceptable representative (LAR) at the same time as the consent for the mother's participation or within 21 days after the infant's birth. We conducted the study (ClinicalTrials.gov identifier NCT03614676) in accordance with the International Council for Harmonisation guidelines for Good Clinical Practice and followed the principles of the Declaration of Helsinki and applicable local guidelines. The study protocol and amendments (available at:https://www.gsk-studyregister.com/en/trial-details/?id=207636) were approved by the relevant institutional review boards or independent ethics committees for each site (Supplementary Materials).

### LRTI Surveillance

**Figure 2.**
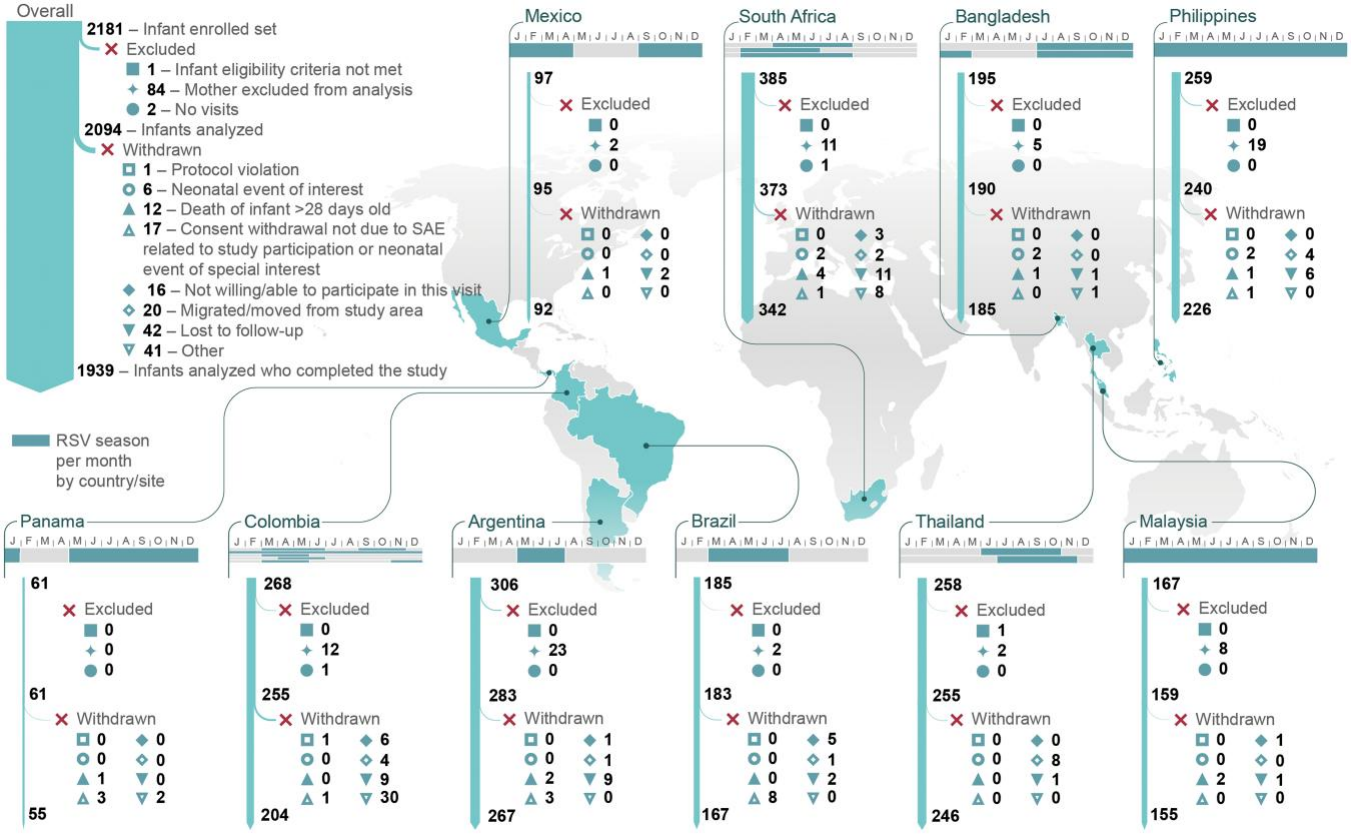
Participant flow diagram. Respiratory syncytial virus (RSV) seasons were defined as described before the coronavirus disease 2019 pandemic and are shown per country and study site (if the transmission season varied for the different sites). Months with RSV transmission for 1 calendar year (from January [J] to December [D]) are highlighted. Information on how the seasons were determined for each site is provided in the Supplementary Materials. Abbreviations: RSV, respiratory syncytial virus; SAE, serious adverse event.

LRTI surveillance was done through active and passive contacts starting at birth and ending at the year 1 visit (Figure 1). In active contacts, site staff contacted the infant's parent(s), LAR(s), or their designate(s) at regular intervals by phone calls, text message, or other means, depending on best local practice. In countries with seasonal RSV transmission (Figure 2; Supplementary Materials: “RSV Seasonality”), this was approximately weekly during the RSV season and monthly during interseason periods. In countries with year-round RSV transmission (Figure 2), active contacts occurred approximately every 2 weeks. During these contacts, the site staff assessed whether the infant had developed (new) LRTI symptoms, that is, cough, runny nose, or blocked nose (respiratory tract illness [RTI] symptoms) together with difficulty breathing, wheezing, or parental concern (Figure 1). In passive contacts (which occurred throughout the year independently of the RSV season), the infant's parent(s), LAR(s), or their designate(s) spontaneously contacted the site staff whenever the infant developed (new) RTI symptoms, difficulty breathing, or wheezing; if the infant's symptoms worsened; or if there was parental concern (Figure 1). During each surveillance contact, the staff reminded the parent(s), LAR(s), or designate(s) to report any future new/worsening RTI symptoms.

If there was difficulty breathing, wheezing, or parental concern, a visit to assess potential LRTI was conducted (Figure 1). During this visit, the site staff evaluated clinical signs and symptoms of the RTI and recorded the infant's temperature, respiratory rate, blood oxygen saturation, signs of difficulty breathing (including wheezing, tachypnea, nasal flaring, chest indrawing, or apnea), and possible presence of COVID-19 infection (Figure 1). Case definitions for any, severe, and very severe RSV-LRTI, RSV hospitalization, and all-cause LRTI were based on those proposed by the WHO RSV Vaccine Consultation Expert Group [20] (Table 1), with LRTI defined by history of cough or difficulty breathing, and blood oxygen saturation <95% or increased respiratory rate; chest X-rays were not required.

**Table 1.** Case Definitions for Respiratory Tract Illness and Lower Respiratory Tract Illness Based on Those Proposed by the World Health Organization Respiratory Syncytial Virus Vaccine Consultation Expert Group [20]

Variable	Case Definition
RSV-RTI	Runny nose or blocked nose or cough AND confirmed RSV infection^a^
RSV-LRTI	History of cough or difficulty breathing^b^ AND SpO_2_ <95%^c^ or RR increase^d^ AND confirmed RSV infection^a^
Severe RSV-LRTI	Meeting the case definition of RSV-LRTI AND SpO_2_ <93%^c^ or lower chest wall indrawing
Very severe RSV-LRTI	Meeting the case definition of RSV-LRTI AND SpO_2_ <90%^c^ or inability to feed or failure to respond/being unconscious
RSV hospitalization	Confirmed RSV infection^a^ AND hospitalized for acute medical condition^e^
All-cause RTI	Runny nose or blocked nose or cough
All-cause LRTI	History of cough or difficulty breathing^b^ AND SpO_2_ <95%^c^ or RR increase^d^

Abbreviations: LRTI, lower respiratory tract illness; RR, respiratory rate; RSV, respiratory syncytial virus; RTI, respiratory tract illness; SpO_2_, blood oxygen saturation measured by pulse oximetry.

^a^RSV infection confirmed on nasal swab positive for RSV-A or RSV-B subtypes by quantitative reverse-transcription polymerase chain reaction.

^b^Based on history reported by parent(s)/legally acceptable representatives; difficulty breathing as evident from, eg, signs of wheezing or stridor, tachypnea, flaring of nostrils, chest indrawing, or apnea.

^c^For SpO_2_, the lowest monitored value was used. At high altitudes (>2500 m), SpO_2_ <92% for LRTI, <90% for severe LRTI, and <87% for very severe LRTI.

^d^RR increase was defined as >60 breaths/minute (<2 months of age), >50 breaths/minute (2–11 months of age), and >40 breaths/minute (12–24 months of age).

^e^Hospitalization was defined as admission for observation or treatment based on the judgment of a healthcare provider.

A nasal swab (Supplementary Materials) was taken for detection of the 2 RSV subtypes (RSV-A and RSV-B) using quantitative reverse-transcription polymerase chain reaction (PCR) (at GSK or a designated laboratory), as previously described [22]. RSV-positive swabs (defined as 304 copies/mL for RSV-A and 475 copies/mL for RSV-B) were also tested for the presence of 17 other respiratory viral types and subtypes using multiplex PCR (Allplex Respiratory Panel [Seegene] or similar). RSV-negative swabs were not tested for other viruses.

The infant's parent(s), LAR(s), or designate(s) recorded RTI symptoms (including start and end dates) in paper diary cards (Supplementary Materials). Appropriate clinical care was provided at the study site or another treating clinical facility per local standard of care. If an infant was admitted to the hospital for an acute medical event and RSV infection was suspected (based on the RTI case definition), a nasal swab was collected (if possible) and tested for RSV-A/B at GSK or a designated laboratory. As the study took place during the COVID-19 pandemic, special measures were implemented (Supplementary Materials).

### Cord Blood Antibodies

Approximately 5–10 mL of cord blood was collected at delivery, and serum RSV-A and RSV-B neutralizing antibodies were measured using an in-house neutralization assay (Supplementary Materials).

### Statistical Analyses

We aimed to enroll approximately 2300 pregnant women and their infants (200–300 per country). Sample size calculations were based on the primary objectives (reported elsewhere). Statistical analyses were performed using SAS software version 9.4 (SAS Institute, Cary, North Carolina). All analyses were performed on infants who met all eligibility criteria up to the time of their censoring (study completion or drop-out) with at least 1 timepoint evaluation and born to enrolled mothers who met all eligibility criteria up to the time of their censoring.

Incidence analyses for RSV-LRTI, severe and very severe RSV-LRTI, RSV hospitalizations, and all-cause LRTI were performed overall, by country, by age stratum (0–2, 0–5, and 0–11 months), and by 1-month intervals. IRs for first episodes (expressed per 100 person-years [PY]) were calculated with exact 95% confidence intervals (CIs) by dividing the number of infants with a first episode during the follow-up by the total PY. Proportions affected were calculated with exact 95% CIs as the number of infants with at least 1 episode within an age interval (0–2, 0–5, and 0–11 months) over the total number of infants at the start of the age interval. Incidence proportions were calculated with exact 95% CIs as the number of infants with a first episode within an age interval (monthly) over the population at risk at the start of the age interval. The person-time at risk for an event was calculated as the time between the date of birth and the end of the at-risk period or the earliest of the following: first diagnosis of the event, first birthday, death, or last follow-up.

Percentages of infants with RSV-LRTI coinfected with other respiratory viruses were calculated with exact 95% CIs and geometric mean titers (GMTs) for RSV-A and RSV-B neutralizing antibodies with 95% CIs.

Missing data were not replaced.

## RESULTS

### Participants

In total, 2181 infants were enrolled and 2094 were included in the analysis, of whom 1939 completed the study. The main reason for withdrawal was loss to follow-up (Figure 2).

The mean gestational age of enrolled infants at birth was 38.5 weeks, and 141 infants (6.7%) were born prematurely (<37 weeks’ gestation). Nearly all infants (97.7%) were breastfed (mean duration, 9.9 months) (Table 2). Demographic characteristics were similar across the different countries (Supplementary Table 1).

**Table 2.** Baseline Characteristics of Participating Infants (Enrolled Set)

Characteristic	Overall (N = 2181)
Gestational age at birth, wk	
Mean (SD)	38.5 (1.7)
Median (range)	39.0 (27–43)
Missing, No.	11
Sex	
Female	1049 (48.1)
Male	1132 (51.9)
Born in RSV transmission season^a^	
Yes	1076 (49.3)
No	1105 (50.7)
Length, cm	
Mean (SD)	49.2 (2.7)
Missing, No.	59
Birth weight, kg	
Mean (SD)	3.1 (0.5)
Missing, No.	29
5-min Apgar score	
Mean (SD)	9.4 (0.8)
Median	9.0
Missing, No.	172
Breastfeeding	
Yes	2131 (97.7)
No	12 (0.6)
Missing, No.	38
Mean duration (SD), mo	9.9 (3.8)

Unless otherwise indicated, data are presented as number (%) of infants in a given category/total number of infants in the enrolled set.

Abbreviations: RSV, respiratory syncytial virus; SD, standard deviation.

^a^RSV transmission season depended on the country and the study site and could include autumn/winter months or rainy seasons or be year-round. For the study, seasons were defined as described before the coronavirus disease 2019 (COVID-19) pandemic (Figure 2; Supplementary Materials: “RSV seasonality”). However, RSV seasonality was impacted by the COVID-19 pandemic [6–8].

Of all enrolled infants, 49.3% were born during the RSV transmission season (with seasons as described before the COVID-19 pandemic; Figure 2), but this proportion varied widely between countries (from 7.0% in Thailand to 100% in Malaysia and the Philippines) depending on the individual country's RSV seasonality (Supplementary Table 1).

### Overall Incidence of RSV-LRTI, RSV Hospitalization, and All-Cause LRTI

**Figure 3.**
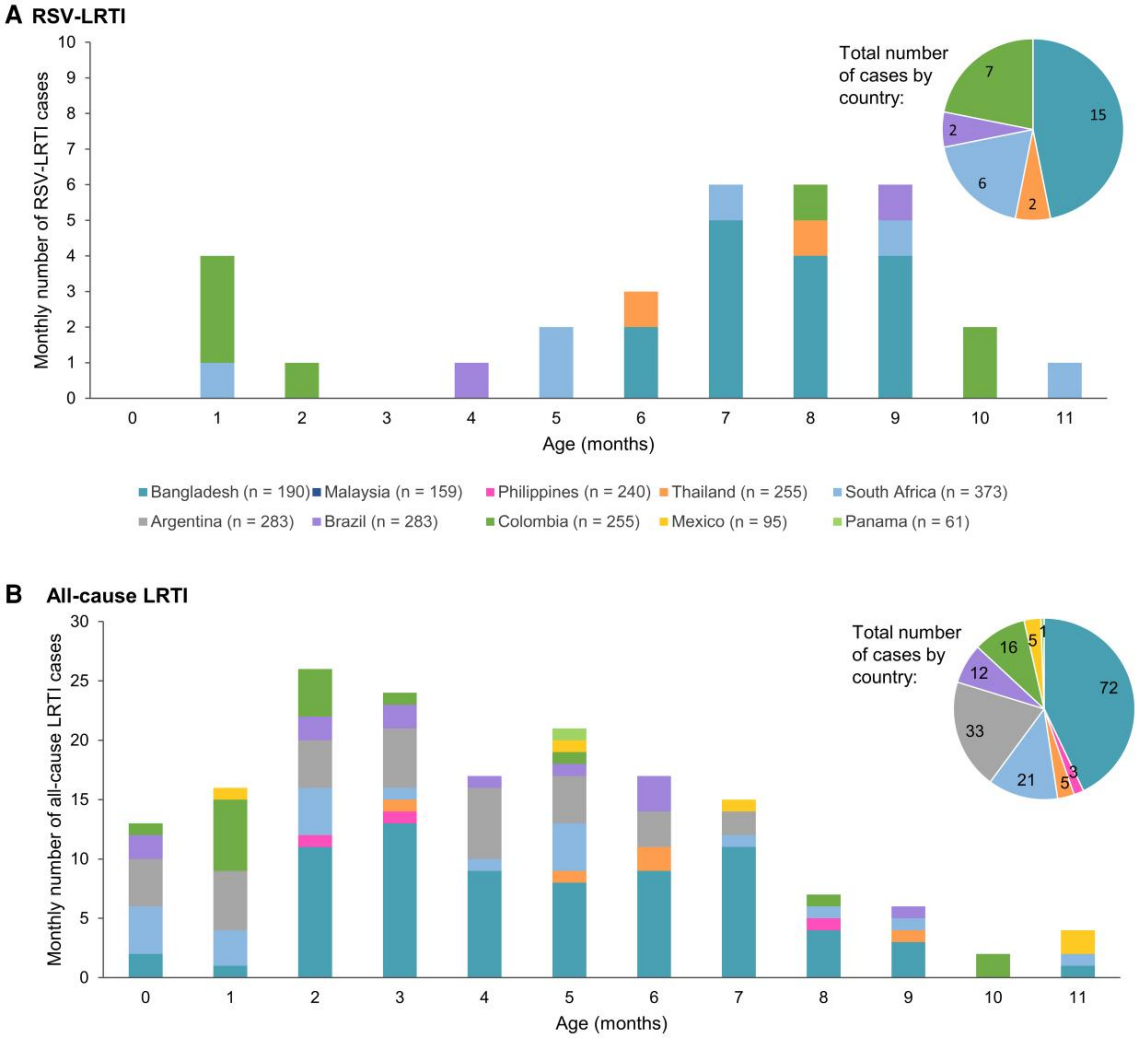
Number of respiratory syncytial virus (RSV)–associated lower respiratory tract illness (LRTI) cases (*A*) and all-cause LRTI cases (*B*) by country and month of life. LRTI is based on the World Health Organization case definition. The n values indicate the total number of infants in the analysis for each country.

**Figure 4.**
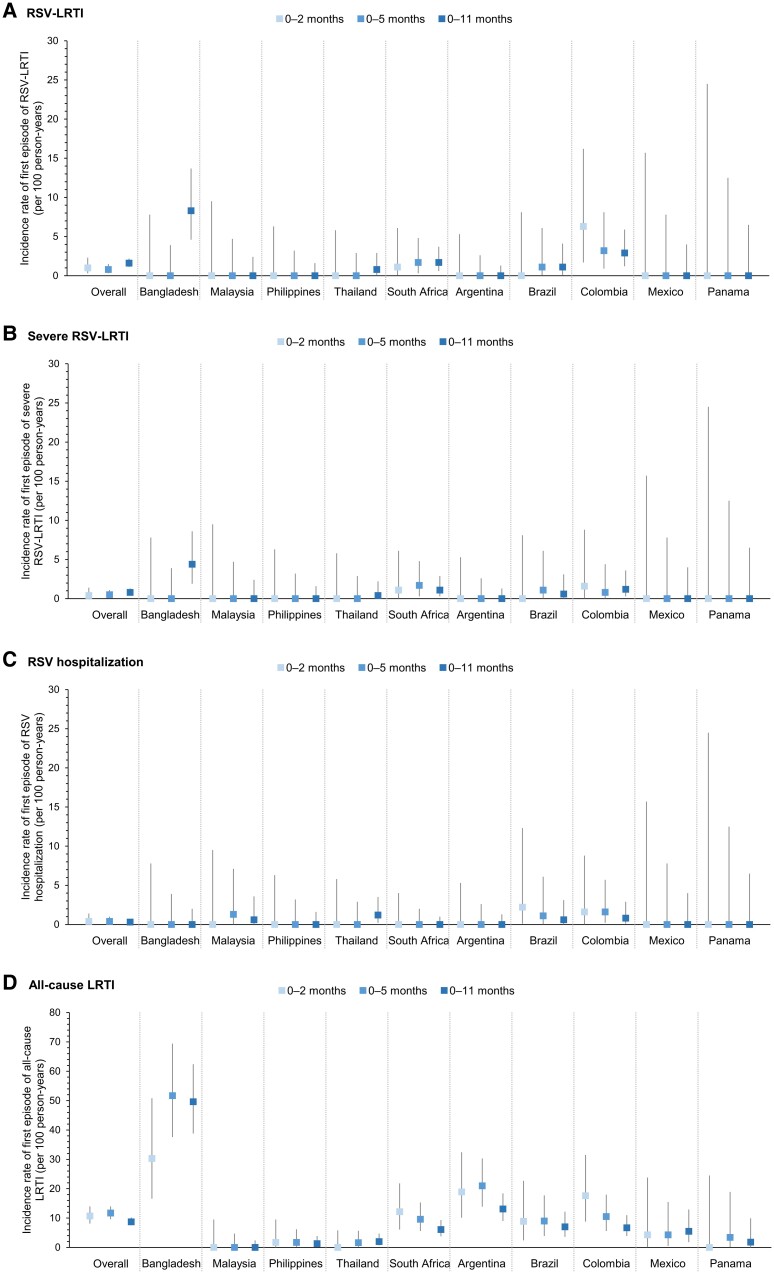
Incidence rates of first episode of respiratory syncytial virus (RSV)–associated lower respiratory tract illness (LRTI) (*A*), severe RSV-LRTI (*B*), RSV hospitalization (*C*), and all-cause LRTI (*D*) by age interval, overall and for each country. Error bars indicate 95% confidence intervals. LRTI is based on the World Health Organization case definition.

Among the 2094 infants included in the analysis, 32 experienced an RSV-LRTI during the 1-year follow-up. No recurrent episodes were reported. Eight infants had an episode during their first 6 months of life; 24 had an episode thereafter (Figure 3). The proportion of infants affected by RSV-LRTI was low (0.2%, 0.4%, and 1.5% for infants aged 0–2, 0–5, and 0–11 months, respectively) (Table 3). The overall IR of first RSV-LRTI episode was 1.0/100 PY (95% CI, .3–2.3) in 0- to 2-month-olds, 0.8/100 PY (95% CI, .3–1.5) in 0- to 5-month-olds, and 1.6/100 PY (95% CI, 1.1–2.2) in 0- to 11-month-olds (Table 3; Figure 4).

**Table 3.** Incidence Rates of First Episodes of Respiratory Syncytial Virus (RSV)–Associated Lower Respiratory Tract Illness (LRTI), Severe RSV-LRTI, RSV Hospitalization, and All-Cause LRTI and Proportions Affected by at Least 1 Episode, Overall by Age Interval

Outcome and Age Group	No. of Infants With a First Episode	Overall (N = 2094)		
		Total PY	Incidence Rate (95% CI), per 100 PY	Proportion Affected, % (95% CI)
RSV-LRTI				
0–2 mo	5	516.7	1.0 (.3–2.3)	0.2 (.1–0.6)
0–5 mo	8	1029.7	0.8 (.3–1.5)	0.4 (.2–.8)
0–11 mo	32	2013.1	1.6 (1.1–2.2)	1.5 (1.0–2.2)
Severe RSV-LRTI				
0–2 mo	2	517.1	0.4 (.0–1.4)	0.1 (.0–.3)
0–5 mo	5	1030.8	0.5 (.2–1.1)	0.2 (.1–.6)
0–11 mo	17	2018.7	0.8 (.5–1.3)	0.8 (.5–1.3)
RSV hospitalization				
0–2 mo	2	516.9	0.4 (.0–1.4)	0.1 (.0–.3)
0–5 mo	4	1030.7	0.4 (.1–1.0)	0.2 (.1–.5)
0–11 mo	7	2022.6	0.3 (.1–.7)	0.3 (.1–.7)
All-cause LRTI				
0–2 mo	55	511.8	10.7 (8.1–14.0)	2.6 (2.0–3.4)
0–5 mo	117	1004.3	11.7 (9.6–14.0)	5.6 (4.6–6.7)
0–11 mo	168	1924.6	8.7 (7.5–10.2)	8.0 (6.9–9.3)

Abbreviations: CI, confidence interval; LRTI, lower respiratory tract illness based on the World Health Organization case definition; PY, person-years; RSV, respiratory syncytial virus.

Seventeen infants had a severe RSV-LRTI: 5 infants in the first 6 months and 12 thereafter. The proportions of infants affected by at least 1 severe RSV-LRTI were 0.1%, 0.2%, and 0.8% for those aged 0–2, 0–5, and 0–11 months, respectively (Table 3). The overall IR of first severe RSV-LRTI episode was 0.4/100 PY (95% CI, .0–1.4) in 0- to 2-month-olds, 0.5/100 PY (95% CI, .2–1.1) in 0- to 5-month-olds, and 0.8/100 PY (95% CI, .5–1.3) in 0- to 11-month-olds (Table 3; Figure 4).

None of the infants experienced a very severe RSV-LRTI.

Seven infants were hospitalized with RSV. The proportions of infants affected by RSV hospitalization among those aged 0–2, 0–5, and 0–11 months were 0.1%, 0.2%, and 0.3%, respectively (Table 3). The IR of first-time RSV hospitalization was comparable in the 3 age strata: 0.4 (95% CI, .0–1.4), 0.4 (95% CI, .1–1.0), and 0.3 (95% CI, .1–.7) per 100 PY (Table 3; Figure 4).

In total, 168 infants experienced at least 1 all-cause LRTI, most (117) of whom had a first episode during their first 6 months of life (Figure 3). There were 37 recurrent all-cause LRTI episodes. The proportions affected by at least 1 all-cause LRTI were 2.6%, 5.6%, and 8.0% for infants aged 0–2, 0–5, and 0–11 months, respectively (Table 3). IRs of first all-cause LRTI episode were 10.7/100 PY (95% CI, 8.1–14.0) in 0- to 2-month-olds, 11.7/100 PY (95% CI, 9.6–14.0) in 0- to 5-month-olds, and 8.7/100 PY (95% CI, 7.5–10.2) in 0- to 11-month-olds (Table 3; Figure 4). Overall, 19.0% of first all-cause LRTI episodes were positive for RSV.

Monthly incidence proportions were relatively homogeneous during the first year of life (0.0%–0.3% for RSV-LRTI, 0.0%–0.2% for severe RSV-LRTI, 0.0%–0.05% for RSV hospitalizations, and 0.1%–1.3% for all-cause LRTI).

### Incidence of RSV-LRTI, RSV Hospitalization, and All-Cause LRTI by Country

No RSV-LRTI cases were reported in infants in Malaysia, the Philippines, Argentina, Mexico, or Panama. Of the 32 RSV-LRTI episodes in 0- to 11-month-olds, 15 were reported in Bangladesh (of which 8 were severe), 7 in Colombia (3 severe), 6 in South Africa (4 severe), and 2 each in Brazil and Thailand (1 severe each). In Bangladesh and Thailand, all cases were reported after the first 6 months of life, whereas in the other countries, cases were spread more uniformly in the first and second 6 months (Figure 3). The IRs of first RSV-LRTI and first severe RSV-LRTI episode during the first year of life were highest in Bangladesh (8.3/100 PY for RSV-LRTI, 4.4/100 PY for severe RSV-LRTI) and varied across the other countries with reported cases (0.8–2.9/100 PY for any RSV-LRTI; 0.4–1.2/100 PY for severe RSV-LRTI) (Figure 4; Supplementary Table 2).

There were no RSV hospitalizations among infants in Bangladesh, the Philippines, South Africa, Argentina, Mexico, or Panama. In the other countries, IRs for first RSV hospitalizations in 0- to 11-month-olds were 0.6–1.2/100 PY (Figure 4; Supplementary Table 2).

All-cause LRTI cases were reported in all countries except Malaysia. Of the 168 first all-cause LRTIs, 72 occurred in infants in Bangladesh (proportion of infants affected: 37.9%), 33 in Argentina (11.7%), 21 in South Africa (5.6%), 16 in Colombia (6.3%), 12 in Brazil (6.6%), 5 each in Thailand and Mexico (2.0% and 5.3%), 3 in the Philippines (1.3%), and 1 in Panama (1.6%). In most countries, more cases were reported in the first 6 months of life (Figure 3; Supplementary Table 2). The highest IR for first all-cause LRTI episode in 0- to 11-month-olds was reported for Bangladesh (49.6/100 PY). IRs varied between 1.3 and 13.1/100 PY for the other countries that had reported cases (Figure 4; Supplementary Table 2). The proportion of first all-cause LRTI episodes that tested positive for RSV ranged from 0.0% to 43.8% across countries.

### Frequency of Symptoms in Infants With RSV-LRTI and All-Cause LRTI

Among the 32 infants with RSV-LRTI, the most frequently reported symptoms were cough (100%), runny nose (81.3%), wheezing (56.3%), and blocked nose (50.0%) (Supplementary Table 3). Among the 168 infants with all-cause LRTI, the most common symptoms during their first episode were cough (92.9%), runny nose (77.4%), blocked nose (65.5%), and wheezing (39.3%) (Supplementary Table 3). Similar frequencies of symptoms were reported during the 37 recurrent episodes (Supplementary Table 3). When empirically comparing all RSV-LRTI with all RSV-negative LRTI cases, the frequency of the different symptoms was generally similar, although blocked nose tended to be more common in RSV-negative LRTI (69.2%) than in RSV-LRTI (50.0%), while the opposite was seen for wheezing (39.5% in RSV-negative LRTI vs 56.3% in RSV-LRTI) and fever (14.0% in RSV-negative LRTI vs 25.0% in RSV-LRTI) (Supplementary Table 3).

### Coinfections of RSV-LRTI With Other Respiratory Viruses

In total, 33 swabs were available from the 32 infants with RSV-LRTI and 16 swabs from the 17 infants with severe RSV-LRTI. Viral coinfection was detected in 13 (39.4%) and 6 (37.5%) of these swabs, respectively, with rhinovirus identified most frequently (Supplementary Table 4).

### Neutralizing RSV Antibodies in Cord Blood of Infants Developing RSV-LRTI

The cord blood samples of all infants, including the 8 infants who developed RSV-LRTI during their first 6 months of life, were seropositive for both RSV-A and RSV-B neutralizing antibodies. GMTs among these 8 infants were 478.2 estimated dilution 60 (ED_60_) (95% CI, 215.9–1059.1) for RSV-A and 697.5 ED_60_ (95% CI, 332.3–1464.3) for RSV-B. Among the 5 infants with RSV-LRTI in the first 3 months of life, GMTs were 322.0 ED_60_ (95% CI, 124.7–831.4) for RSV-A and 543.9 ED_60_ (95% CI, 162.2–1823.4) for RSV-B. This compared to GMTs of 751.5 ED_60_ (95% CI, 724.7–779.4) for RSV-A and 1221.1 ED_60_ (95% CI, 1172.2–1272.1) for RSV-B across all cord blood samples.

The association between cord blood antibodies and RSV-LRTI could not be analyzed because of the low number of RSV-LRTI cases.

### COVID-19 Cases

Throughout the study, 116 COVID-19 cases were diagnosed in 98 (4.5%) enrolled infants. Fifteen infants had multiple COVID-19 diagnoses. Most cases were reported in Brazil (47 cases in 39 infants) and Panama (20 cases in 15 infants) (Supplementary Table 5). Among the 116 reported COVID-19 diagnoses, 16 (13.8%) were confirmed, 24 (20.7%) were probable, and 76 (65.5%) were suspected cases; 108 cases (93.1%) were symptomatic. Of the 16 confirmed COVID-19 cases, 2 had an LRTI (RSV negative).

## DISCUSSION

This prospective study conducted during the COVID-19 pandemic in 10 LMICs showed a relatively low incidence of RSV-LRTI (1.6/100 PY), severe RSV-LRTI (0.8/100 PY), and RSV hospitalization (0.3/100-PY) and an absence of very severe RSV-LRTI in infants during their first year of life. IRs for both RSV-LRTI and severe RSV-LRTI varied widely across countries, with the highest rates in Bangladesh and no cases in Malaysia, the Philippines, Argentina, Mexico, and Panama. Across different countries, RSV-LRTI cases were either reported exclusively after the first 6 months of life or relatively uniformly in the first and second 6 months.

Our estimates were lower than those reported previously [5, 21–24]. A global meta-analysis based on pre-COVID-19 data estimated RSV-LRTI IRs in infants 0–12 months of 7.8/100 PY in low-income, 11.1/100 PY in lower-middle-income, and 10.9/100 PY in upper-middle-income countries [5]. In a study similar to ours conducted from 2013 to 2017, the IRs per 100 PY of RSV-LRTI during the first year of life were higher than those observed in the current study for the same countries (Argentina: 14.0 vs 0.0 in the current study; Bangladesh: 20.3 vs 8.3; South Africa: 3.1 vs 1.7; and Thailand: 2.5 vs 0.8) [24]. The lower IRs in our study are likely explained by NPIs (instated to curb the spread of COVID-19) reducing the circulation of RSV and other respiratory viruses. This was shown in other studies, including in several of the countries in our study [6–10, 25–29]. The impact of NPIs on RSV circulation and seasonality may also explain why in some countries, all RSV-LRTI cases were observed in infants ≥6 months old, while previous studies showed that the greatest burden was during the first 6 months of life [5, 21, 22]. This is consistent with other studies showing that RSV-LRTI cases occurred at an older age during the COVID-19 pandemic compared to before [12, 17]. Delays in the RSV season because of COVID-19–related NPIs and subsequent relaxation of NPIs may have resulted in an expanded group of older RSV-naïve infants [30].

IRs for all-cause LRTI were also lower in our study compared to those reported before the COVID-19 pandemic [24, 31–33]. In the study similar to ours conducted from 2013 to 2017, all-cause LRTI IRs per 100 PY in the first year of life were 40.2 in Argentina (vs 13.1 in our study), 74.0 in Bangladesh (vs 49.6), 14.5 in South Africa (vs 6.1), and 8.1 in Thailand (vs 2.0) [24]. Lower all-cause LRTI rates during versus before COVID-19 have been observed previously [25, 34]. In our study, countries with a higher incidence of all-cause LRTI had a higher incidence of RSV-LRTI. Argentina was an exception, with the 1-but-highest incidence of all-cause LRTI but no RSV-LRTI cases. Contrary to RSV infections, we found that most first episodes of all-cause LRTI occurred during the first 6 months of life. It was shown previously that not all respiratory pathogens were controlled to the same extent by NPIs, possibly due to differences in viral tropism, mode of transmission, sensitivity to disinfectants, and virus–virus interactions [8–10, 35, 36]. Some of the LRTIs may have been caused by COVID-19.

Rhinovirus was identified most often as a second virus in infants with RSV-LRTI, in line with previous studies analyzing viral coinfections in infants and young children with LRTI [37–40]. Rhinovirus was also 1 of the respiratory viruses that was least affected by NPIs and became predominant in several countries during 2020–2021 [8, 10, 35, 36].

Comparing the clinical features of RSV-LRTI cases between our study and other studies is challenging because of differences in age, settings, study periods, and geographic locations [41–46]. However, despite variability in the frequencies of symptoms, the range of symptoms associated with RSV-LRTI was similar across studies, with cough and runny nose being the most common and a substantial proportion of infants presenting wheezing.

While we could not formally analyze the association between cord blood antibodies and RSV-LRTI because of the low number of RSV-LRTI cases, RSV neutralizing antibody GMTs in cord blood of infants who developed RSV-LRTI were lower than in the overall study population, in line with previous observations showing that RSV neutralizing antibodies at birth predict protection against RSV illness [47].

Because our study overlapped with the first years of the COVID-19 pandemic, when NPIs impacted RSV circulation and seasonality, our findings most likely do not reflect a standard RSV season and are applicable only to the countries/regions included in the study in the context of a global pandemic. The small number of cases prevented further subgroup analyses. Another limitation is that our results may not reflect incidences in a high-risk population because the number of high-risk infants was low.

Strengths of our study include the use of active and passive surveillance and standardized case definitions and its focus on LMICs, where the RSV burden is the highest [5], incidence data are sparse, and surveillance programs and the application of harmonized case definitions are often lacking [18, 20].

In conclusion, the RSV-LRTI IRs in the first year of life reported in our study were lower than what was reported before the COVID-19 pandemic, likely due to the impact of NPIs on viral circulation.

## Supplementary Material

ofad553_Supplementary_Data

## Supplementary Data

Supplementary materials are available at *Open Forum Infectious Diseases* online. Consisting of data provided by the authors to benefit the reader, the posted materials are not copyedited and are the sole responsibility of the authors, so questions or comments should be addressed to the corresponding author.

## Notes

***Acknowledgments.*** We are grateful to all participants, their caregivers, study nurses, coordinators, investigators, and other study team members. Medical writing (by Natalie Denef), design, and coordination support were provided by Akkodis Belgium c/o GSK.

***Author contributions.*** R. L., U. D. A. N., A. G., P. D., K. Z., C. E. R., B. M., T. D. L., K. A., O. H., G. D. S., A. N. T., and Y. P. were involved in the conception or the design of the study. M. F. R., A. C., M. D. B., R. L., U. D. A. N., A. G., M. F. L., M. T. R. B., J. C., A. F., P. D., K. Z., M. M. M. P., J. P., A. V. S., C. M. C., J. A. V. P., D. A. A. F., C. E. R., E. L. M., B. M., J. N. R. N., A. S. G., L. S., C. M. F. L., R. S., S. W. T., E. C. D. S., I. H. T., C. d. C. F. A., J. C. T., I. A. d. R., O. R., J. B. V. M., S. K., S. F., K. A., M. M., T. P., L. A., K. C., E. Y., G. D. S., J. H. K., A. N. T., and Y. P. participated in the collection or generation of the study data. M. F. R., A. C., M. D. B., R. L., U. D. A. N., A. G., M. F. L., M. T. R. B., J. C., A. F., P. D., K. Z., M. M. M. P., J. P., A. V. S., C. M. C., C. E. R., E. L. M., B. M., J. N. R. N., A. S. G., L. S., C. M. F. L., R. S., S. W. T., E. C. D. S., I. H. T., C. d. C. F. A., J. C. T., I. A. d. R., O. R., J. B. V. M., S. K., S. F., K. A., M. M., T. P., L. A., K. C., E. Y., G. D. S., J. H. K., Y. P., and A. A. performed the study. A. C., M. D. B., R. L., U. D. A. N., A. G., M. T. R. B., J. C., A. F., P. D., K. Z., J. P., A. V. S., J. N. R. N., A. S. G., I. H. T., I. A. d. R., E. Y., Y. P., and A. A. contributed to the study with materials/analysis tools. R. L., A. G., P. D., K. Z., J. N. R. N., J. C. T., I. A. d. R., K. C., E. Y., G. D. S., J. H. K., S. P., A. N. T., Y. P., and A. A. were involved in the analysis or interpretation of the data. All authors approved the manuscript.

***Data availability.*** Study data and documents can be requested for further research from www.clinicalstudydatarequest.com.

***Financial support.*** This work was supported by GlaxoSmithKline Biologicals SA.
