# Supplementary material for: Incidence of Respiratory Syncytial Virus–Associated Lower Respiratory Tract Illness in Infants in Low- and Middle-Income Regions During the Coronavirus Disease 2019 Pandemic
Source: Open Forum Infect Dis. 2023 Dec 1;10(12):ofad553. doi: 10.1093/ofid/ofad553 (PMC10715683; doi:10.1093/ofid/ofad553)
Supplement: ofad553_Supplementary_Data [file ofad553_supplementary_data.pdf]

Supplementary material

Inclusion criteria

All participants had to satisfy all the following criteria at study entry:

Pregnant women

- Healthy pregnant women 18–45 years of age who were $\geq 24^{0/7}$ weeks gestational age at screening and $\leq 27^{6/7}$ weeks gestational age at visit 1, as established by ultrasound examination and/or last menstrual period date.
    - The level of diagnostic certainty of the gestational age had to be established by using the Global Alignment of Immunization Safety Assessment in Pregnancy gestational age assessment form.
- Women with pre-pregnancy body mass index ≥18.5 and ≤39.9 kg/m^2.
- Women whose pregnancy was considered low risk, based on medical history, obstetric history, and clinical findings during the current pregnancy.
- Women who had no significant findings (such as abnormal fetal morphology, amniotic fluid levels, placenta or umbilical cord) observed during a level 2 ultrasound (fetal morphology assessment).
- Human immunodeficiency virus (HIV)-uninfected women who had been tested within the past year and had documented HIV-negative test results.
- Women who gave written or witnessed/thumb printed informed consent after the study had been explained according to local regulatory requirements.
    - The informed consent given at screening had to either include consent for both the mother's participation and participation of the infant after the infant's birth (if consistent with local regulations/guidelines), or consent for the mother's participation and expressed willingness to consider permitting the infant to take part

after the infant was born (if local regulations/guidelines required parent(s) to provide an additional informed consent after the infant's birth).
    - Both mother and father had to consent if local regulations/guidelines required it.
- Individuals who consented to have cord blood collected at delivery for the purpose of the study.
- Individuals who planned to reside in the study area for at least one year after delivery.
- Individuals who were in good health as determined by the outcome of medical history, physical examination and clinical judgment of the investigator.
- Individuals who, in the opinion of the investigator, could and would comprehend and comply with all study procedures (e.g., return for study follow-up visits, be contactable and available on a regular basis for surveillance).

Infants

- Infants who were in utero at the time maternal (and paternal, if required) informed consent was given, and who were live born.
- If required by local law: written or witnessed/thumb printed informed consent for study participation of the infant was obtained from parent(s)/legally acceptable representative(s) (LARs) within 21 days of birth.

Exclusion criteria

The following criteria had to be checked at the screening visit and at visit 1. If any exclusion criterion applied, the individual could not be included in the study.

Pregnant women

- Women determined to have one of the following conditions associated with increased risk for a serious obstetrical complication:
    - Gestational hypertension

    - Gestational diabetes uncontrolled by diet and exercise
    - Pre-eclampsia or eclampsia
    - Multiple pregnancy
    - Intrauterine growth restriction
    - Placenta previa
    - Polyhydramnios
    - Oligohydramnios
- Women determined to have (during the current pregnancy) one of the following infections or conditions associated with risk of adverse outcome:
    - Known or suspected:
        - Syphilis infection
        - Parvovirus B19
        - Rubella infection
        - Primary genital herpes simplex infection
        - Primary cytomegalovirus infection
        - Varicella infection
        - Zika infection
        - Active tuberculosis infection
    - Incompetent cervix or cerclage
- Women who had any underlying condition or infection that would predispose them to increased risk for a serious obstetrical complication not mentioned above.
- Women who had behavioral or cognitive impairment or psychiatric disease that, in the opinion of the investigator, could interfere with the woman's ability to participate in the study.

- Women who had known or suspected impairment of the immune system, an active autoimmune disorder that was not well controlled or who were receiving systemic immunosuppressive therapy.
- Women participating in any concurrent clinical trial during the current pregnancy.
- Women pregnant with a fetus with a confirmed or suspected major congenital anomaly at the time of enrollment.

Infants

- Child in care.

Ethics committees

Argentina

- Instituto Medico Rio Cuarto
- Hospital Luis C. Lagomaggiore Comite de Etica en Investigacion
- Hospital de Ninos 'Ricardo Gutierrez' Comite de Etica en Investigacion
- Gobierno de la Ciudad de Buenos Aires, Hospital General de Agudos J.M. Ramos Mejia Comite de Etica en Investigacion
- Gobierno de la Ciudad de Buenos Aires, Ministerio de Salud, Hospital General de Agudos 'Donacion Francisco Santojanni', Comite de Docencia E Investigacion

Bangladesh

- International Centre for Diarrhoeal Disease Research, Bangladesh (Icddr,b)

Brazil

- USP - Hospital Das Clinicas Da Faculdade De Medicina De Ribeirao Preto Da USP
- Universidade Federal De Minas Gerais
- Universidade Federal De Santa Maria/ Pro- Reitoria De Pos-Graduacao E Pesquisa

Colombia

- Comité De Etica En Investigacion Con Seres Humanos Ceish
- Comité Institucional de Ética en Investigacion en seres Humanos de la Universidad CES
- Comité de ética en Investigación- Hospital Mental de Antioquia
- Corporacion Cientifica Pediatrica Comite De Etica En Investigacion Biomedica
- Comité Corporativo de Ética en Investigación
- Comité de Ética de la Investigacion Fundación Hospitalaria San Vicente de Paul

Malaysia

- Jawatankuasa Etika & Penyelidikan Perubatan

Mexico

- Comité de Ética en Investigación de Oaxaca Site Management Organization S.C.
- Comité de Etica en Investigacion, Monterrey
- Comité de Etica en Investigación, Hospital la Mision S.A de C.V.
- Comité de Ética en Investigación del Hospital General De Durango

Panama

- Hospital Del Niño, Dr. José Renán Esquivel

Philippines

- St. Luke's Medical Center Ethics Review Committee
- University of the Philippines Manila Research Ethics Board
- Perpetual Succour Hospital Institutional Ethics and Review Board

South Africa

- Pharma Ethics

- Universiteit Stellenbosch University Health Research Ethics Committee (HREC)

Thailand

- Research Ethics Committee Faculty of Medicine, Chiang Mai University, Research Institute for Health Sciences, Chiang Mai University
- Siriraj Institutional Review Board Faculty of Medicine Siriraj Hospital, Mahidol University Panel 4
- Institutional Review Board, Faculty of Medicine, Chulalongkorn University

RSV seasonality

RSV seasonality by study site was determined as follows:

- Based on data from surveillance reports and peer-reviewed articles, the study sponsor prepared an RSV seasonality table and provided this to the study sites. The RSV season for each site was determined using the information in this table as a guideline. In addition, local surveillance data (regional, provincial, or hospital-based) were reviewed by the site if available and considered reliable by the site investigator. After review, each site proposed an expected duration of the RSV season. The proposed RSV season was shared and approved by the sponsor’s central study team before study start.
- The sponsor’s representative or local study contact worked with each site to agree on the process to determine the start and end dates of the RSV season for RSV surveillance purposes. Determination of the local RSV season had to be based as much as possible on documented data in the subregion of the investigator site (e.g., national-, district-, or provincial-level surveillance data). If official surveillance data were not available, hospital records at the site from previous years could be used to determine the start and end of the RSV season.

- Subregional differences had to be considered for countries where intra-country variation in RSV seasonality exists.

Nasal swabs

Nasal swabs were taken by inserting the cotton bud of the swab to the point of the flocked tip just entering the nostril; the swab was not to be inserted any deeper than 2 cm inside the nostril of the infant. Swabs were rotated gently 180° three times inside the nostril and left in place for 5 seconds before removing and transferring it to the transport tube. Tubes were frozen at -70°C immediately after collection. If that was not possible, tubes could be kept and transported refrigerated between +2°C and +8°C for 12 hours maximum.

Diary cards

Paper infant diary cards were given to the parent(s)/LAR(s)/designate(s) after the infant's birth, and new cards were provided whenever needed during the infant's follow-up.

Parent(s)/LAR(s)/designate(s) were trained by the site on how to complete the diary cards every time a new card was distributed. The diary card also had detailed completion instructions. During the active and passive surveillance contacts, the site staff reminded the parent(s)/LAR(s)/designate(s) to complete the cards in a timely manner. The cards were used to record symptoms for each respiratory tract illness (RTI) episode the infant experienced (one card per episode) and included the start and end dates of each symptom. The end date was the last day the symptom occurred. RTI symptoms listed on the cards were cough, runny nose, blocked nose, wheezing, and difficulty breathing. Symptoms had to be recorded as soon as the infant experienced any of the listed symptoms. The cards were also used to document visits to a pediatric healthcare provider not affiliated with the study, prescription medications (including those taken to treat an RTI), and vaccinations administered to the infant.

Completed diary cards were reviewed by the investigator together with the parent(s)/LAR(s)/designate(s). Cards were returned to the site when cough, blocked nose, difficulty breathing, and wheezing had stopped, and end dates were documented.

Special measures during COVID-19 pandemic

Because the study took place during the COVID-19 pandemic, the following measures were implemented to guarantee the participants' and staff's wellbeing:

- If it was not possible to conduct a protocol-specified, scheduled or event-driven visit (e.g., a visit for lower respiratory tract illness [LRTI] assessment), the visit could be replaced with a contact conducted by telephone, videotelephony or telemedicine. SMS and email were not allowed.
- Biological samples could be collected at a location other than the study site (identified by the investigator and meeting Good Clinical Practice requirements) or at the infant's home. Biological samples were not to be collected if they could not be obtained within the visit interval, processed in a timely manner or appropriately stored until the intended use.
    - Nasal swabs could only be collected using centrally provided supplies.
    - Cord blood could be collected locally but had to be retrieved, processed, and stored in accordance with the Investigator Laboratory Manual.
- Diary cards could be transmitted from and to the site by electronic mail and/or conventional mail.

Neutralization assay

The neutralization assay was performed at GSK or a designated laboratory. A fixed amount of respiratory syncytial virus (RSV)-A strain (Long, ATCC No. VR-26) or RSV-B strain (18537, ATCC No. VR-1580) was incubated with serial dilutions of the test serum. The serum-virus mixture was transferred onto a monolayer of Vero cells (African Green Monkey, kidney, *Cercopitheus aethiops*, ATCC CCL-81) and incubated for 3 days to allow infection of the Vero cells by non-neutralized virus and the

formation of plaques in the cell monolayer. Following a fixation step, RSV-infected cells were detected using anti-RSV immunoglobulin G as primary antibody and a secondary antibody conjugated to horse-radish peroxidase, allowing the visualization of plaques by immunofluorescence after coloration with *TrueBlue* peroxidase substrate. Viral plaques were counted using an automated microscope coupled to an image analyzer (Scanlab system with Axiovision software). For each serum dilution, a ratio, expressed as a percentage, was calculated between the number of plaques at that dilution and the number of plaques in the virus control wells (no serum added). The serum neutralizing antibody titer (expressed in estimated dilution-60 [ED60]) corresponds to the inverse of the interpolated serum dilution that yields a 60% reduction in the number of plaques compared to the virus control wells. Seropositivity cut-offs were 18 ED60 for RSV-A and 30 ED60 for RSV-B.

Supplementary Table 1. Baseline characteristics of participating infants, by country (enrolled set)

Characteristic	Bangladesh N=195	Malaysia N=167	Philippines N=259	Thailand N=258	South Africa N=385	Argentina N=306	Brazil N=185	Colombia N=268	Mexico N=97	Panama N=61
Gestational age at birth										
Mean (SD), weeks	38.2 (1.6)	38.1 (1.9)	38.4 (1.5)	38.4 (1.2)	38.8 (2.0)	38.5 (1.6)	38.9 (1.6)	38.6 (1.6)	38.3 (1.8)	38.9 (1.5)
Median (range), weeks	38.0 (32–42)	38.0 (27–41)	39.0 (28–43)	38.0 (34–41)	39.0 (28–42)	39.0 (28–41)	39.0 (28–42)	39.0 (30–41)	38.0 (29–41)	39.0 (34–41)
Missing, n	0	0	7	0	1	1	0	1	0	1
Sex										
Female, n (%)	87 (44.6)	76 (45.5)	113 (43.6)	123 (47.7)	182 (47.3)	170 (55.6)	89 (48.1)	136 (50.7)	45 (46.4)	28 (45.9)
Male, n (%)	108 (55.4)	91 (54.5)	146 (56.4)	135 (52.3)	203 (52.7)	136 (44.4)	96 (51.9)	132 (49.3)	52 (53.6)	33 (54.1)
Born in RSV transmission season[a]										
Yes, n (%)	21 (10.8)	167 (100)	259 (100)	18 (7.0)	171 (44.4)	89 (29.1)	98 (53.0)	142 (53.0)	59 (60.8)	52 (85.2)
No, n (%)	174 (89.2)	0 (0.0)	0 (0.0)	240 (93.0)	214 (55.6)	217 (70.9)	87 (47.0)	126 (47.0)	38 (39.2)	9 (14.8)
Length										
Mean (SD), cm	47.5 (2.3)	48.6 (2.6)	48.9 (2.7)	49.6 (2.0)	49.8 (3.4)	48.9 (2.5)	49.1 (2.1)	49.8 (2.4)	49.6 (3.0)	50.0 (2.6)
Missing, n	15	0	14	0	26	1	0	2	0	1
Birth weight										
Mean (SD), kg	2.8 (0.4)	3.0 (0.5)	2.9 (0.4)	3.1 (0.4)	3.1 (0.5)	3.3 (0.5)	3.3 (0.5)	3.2 (0.4)	3.1 (0.5)	3.3 (0.4)
Missing, n	12	0	5	0	9	1	0	1	0	1
5-minute Apgar score										
Mean (SD)	9.2 (0.9)	9.4 (0.8)	9.0 (0.2)	9.7 (0.5)	9.6 (0.7)	9.3 (0.8)	9.4 (0.8)	9.4 (1.2)	9.1 (0.5)	9.0 (0.1)

Median	9.0	9.5	9.0	10.0	10.0	9.0	9.0	10.0	9.0	9.0
Missing, n	20	47	23	0	40	3	0	38	0	1
Breastfeeding										
Yes, n (%)	191 (97.9)	162 (97.0)	248 (95.8)	252 (97.7)	372 (96.6)	303 (99.0)	183 (98.9)	267 (99.6)	95 (97.9)	58 (95.1)
No, n (%)	1 (0.5)	1 (0.6)	2 (0.8)	1 (0.4)	3 (0.8)	1 (0.3)	1 (0.5)	0 (0.0)	1 (1.0)	1 (1.6)
Missing, n (%)	3 (1.5)	4 (2.4)	9 (3.5)	5 (1.9)	10 (2.6)	2 (0.7)	1 (0.5)	1 (0.4)	1 (1.0)	2 (3.3)
Mean duration (SD), months	11.5 (2.0)	8.5 (4.7)	8.7 (4.4)	8.7 (4.2)	10.0 (3.7)	11.1 (3.0)	8.8 (4.2)	10.5 (3.2)	10.3 (3.6)	10.9 (3.0)

N, total number of infants in the enrolled set; SD, standard deviation; n (%), number (percentage) of infants in a given category; RSV, respiratory syncytial virus.

[a]RSV transmission season depended on the country and the study site and could include autumn/winter months, rainy seasons or be year-round. For the study, seasons were defined as described before the COVID-19 pandemic (**Figure 2**, **Supplementary material**). However, RSV seasonality was impacted by the COVID-19 pandemic.

Supplementary Table 2. Incidence rates of first episodes of RSV-LRTI, severe RSV-LRTI, RSV hospitalization, and all-cause LRTI and proportions affected by at least one episode by age interval, overall and for each country

Country	0–2 months					0–5 months				0–11 months			
	N	n	p-yr	IR (95% CI), /100 p-yr	PA (95% CI), %	n	p-yr	IR (95% CI), /100 p-yr	PA (95% CI), %	n	p-yr	IR (95% CI), /100 p-yr	PA (95% CI), %
**RSV-LRTI**													
Overall	2094	5	516.7	1.0 (0.3–2.3)	0.2 (0.1–0.6)	8	1029.7	0.8 (0.3–1.5)	0.4 (0.2–0.8)	32	2013.1	1.6 (1.1–2.2)	1.5 (1.0–2.2)
Bangladesh	190	0	47.1	0.0 (0.0–7.8)	0.0 (0.0–1.9)	0	93.8	0.0 (0.0–3.9)	0.0 (0.0–1.9)	15	181.1	8.3 (4.6–13.7)	7.9 (4.5–12.7)
Malaysia	159	0	39.0	0.0 (0.0–9.5)	0.0 (0.0–2.3)	0	78.1	0.0 (0.0–4.7)	0.0 (0.0–2.3)	0	155.1	0.0 (0.0–2.4)	0.0 (0.0–2.3)
Philippines	240	0	58.4	0.0 (0.0–6.3)	0.0 (0.0–1.5)	0	116.1	0.0 (0.0–3.2)	0.0 (0.0–1.5)	0	227.6	0.0 (0.0–1.6)	0.0 (0.0–1.5)
Thailand	255	0	63.2	0.0 (0.0–5.8)	0.0 (0.0–1.4)	0	126.0	0.0 (0.0–2.9)	0.0 (0.0–1.4)	2	247.5	0.8 (0.1–2.9)	0.8 (0.1–2.8)
South Africa	373	1	91.4	1.1 (0.0–6.1)	0.3 (0.0–1.5)	3	181.7	1.7 (0.3–4.8)	0.8 (0.2–2.3)	6	356.2	1.7 (0.6–3.7)	1.6 (0.6–3.5)
Argentina	283	0	70.3	0.0 (0.0–5.3)	0.0 (0.0–1.3)	0	140.3	0.0 (0.0–2.6)	0.0 (0.0–1.3)	0	275.1	0.0 (0.0–1.3)	0.0 (0.0–1.3)
Brazil	183	0	45.6	0.0 (0.0–8.1)	0.0 (0.0–2.0)	1	91.0	1.1 (0.0–6.1)	0.5 (0.0–3.0)	2	177.5	1.1 (0.1–4.1)	1.1 (0.1–3.9)
Colombia	255	4	63.3	6.3 (1.7–16.2)	1.6 (0.4–4.0)	4	126.1	3.2 (0.9–8.1)	1.6 (0.4–4.0)	7	243.7	2.9 (1.2–5.9)	2.7 (1.1–5.6)
Mexico	95	0	23.5	0.0 (0.0–15.7)	0.0 (0.0–3.8)	0	47.0	0.0 (0.0–7.8)	0.0 (0.0–3.8)	0	92.5	0.0 (0.0–4.0)	0.0 (0.0–3.8)
Panama	61	0	15.0	0.0 (0.0–24.5)	0.0 (0.0–5.9)	0	29.6	0.0 (0.0–12.5)	0.0 (0.0–5.9)	0	56.9	0.0 (0.0–6.5)	0.0 (0.0–5.9)
**Severe RSV-LRTI**													
Overall	2094	2	517.1	0.4 (0.0–1.4)	0.1 (0.0–0.3)	5	1030.8	0.5 (0.2–1.1)	0.2 (0.1–0.6)	17	2018.7	0.8 (0.5–1.3)	0.8 (0.5–1.3)

Bangladesh	190	0	47.1	0.0 (0.0–7.8)	0.0 (0.0–1.9)	0	93.8	0.0 (0.0–3.9)	0.0 (0.0–1.9)	8	183.0	4.4 (1.9–8.6)	4.2 (1.8–8.1)
Malaysia	159	0	39.0	0.0 (0.0–9.5)	0.0 (0.0–2.3)	0	78.1	0.0 (0.0–4.7)	0.0 (0.0–2.3)	0	155.1	0.0 (0.0–2.4)	0.0 (0.0–2.3)
Philippines	240	0	58.4	0.0 (0.0–6.3)	0.0 (0.0–1.5)	0	116.1	0.0 (0.0–3.2)	0.0 (0.0–1.5)	0	227.6	0.0 (0.0–1.6)	0.0 (0.0–1.5)
Thailand	255	0	63.2	0.0 (0.0–5.8)	0.0 (0.0–1.4)	0	126.0	0.0 (0.0–2.9)	0.0 (0.0–1.4)	1	247.9	0.4 (0.0–2.2)	0.4 (0.0–2.2)
South Africa	373	1	91.4	1.1 (0.0–6.1)	0.3 (0.0–1.5)	3	181.7	1.7 (0.3–4.8)	0.8 (0.2–2.3)	4	356.6	1.1 (0.3–2.9)	1.1 (0.3–2.7)
Argentina	283	0	70.3	0.0 (0.0–5.3)	0.0 (0.0–1.3)	0	140.3	0.0 (0.0–2.6)	0.0 (0.0–1.3)	0	275.1	0.0 (0.0–1.3)	0.0 (0.0–1.3)
Brazil	183	0	45.6	0.0 (0.0–8.1)	0.0 (0.0–2.0)	1	91.0	1.1 (0.0–6.1)	0.5 (0.0–3.0)	1	177.7	0.6 (0.0–3.1)	0.5 (0.0–3.0)
Colombia	255	1	63.6	1.6 (0.0–8.8)	0.4 (0.0–2.2)	1	127.2	0.8 (0.0–4.4)	0.4 (0.0–2.2)	3	246.4	1.2 (0.3–3.6)	1.2 (0.2–3.4)
Mexico	95	0	23.5	0.0 (0.0–15.7)	0.0 (0.0–3.8)	0	47.0	0.0 (0.0–7.8)	0.0 (0.0–3.8)	0	92.5	0.0 (0.0–4.0)	0.0 (0.0–3.8)
Panama	61	0	15.0	0.0 (0.0–24.5)	0.0 (0.0–5.9)	0	29.6	0.0 (0.0–12.5)	0.0 (0.0–5.9)	0	56.9	0.0 (0.0–6.5)	0.0 (0.0–5.9)
**RSV hospitalization**													
Overall	2094	2	516.9	0.4 (0.0–1.4)	0.1 (0.0–0.3)	4	1030.7	0.4 (0.1–1.0)	0.2 (0.1–0.5)	7	2022.6	0.3 (0.1–0.7)	0.3 (0.1–0.7)
Bangladesh	190	0	47.1	0.0 (0.0–7.8)	0.0 (0.0–1.9)	0	93.8	0.0 (0.0–3.9)	0.0 (0.0–1.9)	0	185.8	0.0 (0.0–2.0)	0.0 (0.0–1.9)
Malaysia	159	0	39.0	0.0 (0.0–9.5)	0.0 (0.0–2.3)	1	78.1	1.3 (0.0–7.1)	0.6 (0.0–3.5)	1	154.6	0.6 (0.0–3.6)	0.6 (0.0–3.5)
Philippines	240	0	58.4	0.0 (0.0–6.3)	0.0 (0.0–1.5)	0	116.1	0.0 (0.0–3.2)	0.0 (0.0–1.5)	0	227.6	0.0 (0.0–1.6)	0.0 (0.0–1.5)
Thailand	255	0	63.2	0.0 (0.0–5.8)	0.0 (0.0–1.4)	0	126.0	0.0 (0.0–2.9)	0.0 (0.0–1.4)	3	247.8	1.2 (0.2–3.5)	1.2 (0.2–3.4)
South Africa	373	0	91.5	0.0 (0.0–4.0)	0.0 (0.0–1.0)	0	182.1	0.0 (0.0–2.0)	0.0 (0.0–1.0)	0	358.7	0.0 (0.0–1.0)	0.0 (0.0–1.0)
Argentina	283	0	70.3	0.0 (0.0–5.3)	0.0 (0.0–1.3)	0	140.3	0.0 (0.0–2.6)	0.0 (0.0–1.3)	0	275.1	0.0 (0.0–1.3)	0.0 (0.0–1.3)
Brazil	183	1	45.4	2.2 (0.1–12.3)	0.5 (0.0–3.0)	1	90.6	1.1 (0.0–6.1)	0.5 (0.0–3.0)	1	177.7	0.6 (0.0–3.1)	0.5 (0.0–3.0)
Colombia	255	1	63.6	1.6 (0.0–8.8)	0.4 (0.0–2.2)	2	127.0	1.6 (0.2–5.7)	0.8 (0.1–2.8)	2	246	0.8 (0.1–2.9)	0.8 (0.1–2.8)

Mexico	95	0	23.5	0.0 (0.0–15.7)	0.0 (0.0–3.8)	0	47.0	0.0 (0.0–7.8)	0.0 (0.0–3.8)	0	92.5	0.0 (0.0–4.0)	0.0 (0.0–3.8)
Panama	61	0	15.0	0.0 (0.0–24.5)	0.0 (0.0–5.9)	0	29.6	0.0 (0.0–12.5)	0.0 (0.0–5.9)	0	56.9	0.0 (0.0–6.5)	0.0 (0.0–5.9)
**All-cause LRTI**													
Overall	2094	55	511.8	10.7 (8.1–14.0)	2.6 (2.0–3.4)	117	1004.3	11.7 (9.6–14.0)	5.6 (4.6–6.7)	168	1924.6	8.7 (7.5–10.2)	8.0 (6.9–9.3)
Bangladesh	190	14	46.2	30.3 (16.6–50.8)	7.4 (4.1–12.1)	44	85.1	51.7 (37.6–69.4)	23.2 (17.4–29.8)	72	145.3	49.6 (38.8–62.4)	37.9 (31.0–45.2)
Malaysia	159	0	39.0	0.0 (0.0–9.5)	0.0 (0.0–2.3)	0	78.1	0.0 (0.0–4.7)	0.0 (0.0–2.3)	0	155.1	0.0 (0.0–2.4)	0.0 (0.0–2.3)
Philippines	240	1	58.4	1.7 (0.0–9.5)	0.4 (0.0–2.3)	2	115.6	1.7 (0.2–6.2)	0.8 (0.1–3.0)	3	225.8	1.3 (0.3–3.9)	1.3 (0.3–3.6)
Thailand	255	0	63.2	0.0 (0.0–5.8)	0.0 (0.0–1.4)	2	125.7	1.6 (0.2–5.7)	0.8 (0.1–2.8)	5	245.8	2.0 (0.7–4.7)	2.0 (0.6–4.5)
South Africa	373	11	90.2	12.2 (6.1–21.8)	2.9 (1.5–5.2)	17	177.6	9.6 (5.6–15.3)	4.6 (2.7–7.2)	21	344.9	6.1 (3.8–9.3)	5.6 (3.5–8.5)
Argentina	283	13	68.7	18.9 (10.1–32.4)	4.6 (2.5–7.7)	28	133.4	21.0 (13.9–30.3)	9.9 (6.7–14.0)	33	252.3	13.1 (9.0–18.4)	11.7 (8.2–16.0)
Brazil	183	4	45.1	8.9 (2.4–22.7)	2.2 (0.6–5.5)	8	89.0	9.0 (3.9–17.7)	4.4 (1.9–8.4)	12	171.3	7.0 (3.6–12.2)	6.6 (3.4–11.2)
Colombia	255	11	62.5	17.6 (8.8–31.5)	4.3 (2.2–7.6)	13	123.4	10.5 (5.6–18.0)	5.1 (2.7–8.6)	16	237.1	6.7 (3.9–11.0)	6.3 (3.6–10.0)
Mexico	95	1	23.4	4.3 (0.1–23.8)	1.1 (0.0–5.7)	2	46.7	4.3 (0.5–15.5)	2.1 (0.3–7.4)	5	90.7	5.5 (1.8–12.9)	5.3 (1.7–11.9)
Panama	61	0	15.0	0.0 (0.0–24.5)	0.0 (0.0–5.9)	1	29.5	3.4 (0.1–18.9)	1.6 (0.0–8.8)	1	56.3	1.8 (0.0–9.9)	1.6 (0.0–8.8)

RSV, respiratory syncytial virus; LRTI, lower respiratory tract illness based on the World Health Organization case definition; N, total number of infants in the analysis; n, number of infants with a first episode; p-yr, person-years; IR, incidence rate; CI, confidence interval; PA, proportion affected.

Supplementary Table 3. Frequencies of symptoms among infants with first and recurrent[a] episodes of RSV-LRTI, all-cause LRTI and RSV-negative LRTI

Symptoms	RSV-LRTI First (=all) episodes[a] (N=32)		All-cause LRTI First episodes (N=168)		All-cause LRTI Recurrent episodes (N=37)		RSV-negative LRTI All episodes (N=172)	
	n	% (95% CI)	n	% (95% CI)	n	% (95% CI)	n	% (95% CI)
Runny nose	26	81.3 (63.6–92.8)	130	77.4 (70.3–83.5)	33	89.2 (74.6–97.0)	136	79.1 (72.2–84.9)
Blocked nose	16	50.0 (31.9–68.1)	110	65.5 (57.8–72.6)	25	67.6 (50.2–82.0)	119	69.2 (61.7–76.0)
Cough	32	100 (89.1–100)	156	92.9 (87.9–96.3)	35	94.6 (81.8–99.3)	158	91.9 (86.7–95.5)
Wheezing	18	56.3 (37.7–73.6)	66	39.3 (31.9–47.1)	20	54.1 (36.9–70.5)	68	39.5 (32.2–47.3)
Grunting	2	6.3 (0.8–20.8)	8	4.8 (2.1–9.2)	0	0.0 (0.0–9.5)	6	3.5 (1.3–7.4)
Nasal flaring	1	3.1 (0.1–16.2)	13	7.7 (4.2–12.9)	5	13.5 (4.5–28.8)	17	9.9 (5.9–15.4)
Intercostal recession	10	31.3 (16.1–50.0)	50	29.8 (23.0–37.3)	11	29.7 (15.9–47.0)	50	29.1 (22.4–36.5)
Temperature ≥38°C	8	25.0 (11.5–43.4)	25	14.9 (9.9–21.2)	7	18.9 (8.0–35.2)	24	14.0 (9.1–20.0)
Chest wall indrawing	0	0.0 (0.0–10.9)	0	0.0 (0.0–2.2)	0	0.0 (0.0–9.5)	0	0.0 (0.0–2.1)
Apnea	0	0.0 (0.0–10.9)	0	0.0 (0.0–2.2)	0	0.0 (0.0–9.5)	0	0.0 (0.0–2.1)

RSV, respiratory syncytial virus; LRTI, lower respiratory tract illness based on the World Health Organization case definition; N, total number of first, recurrent, or all episodes, as indicated; n, number of episodes with the indicated symptom; CI, confidence interval.

[a]No recurrent episodes of RSV-LRTI were reported.

Supplementary Table 4. Percentage of RSV-LRTI and severe RSV-LRTI cases co-infected with other respiratory viruses during the first year of life, overall and by respiratory virus

Respiratory virus identified	RSV-LRTI N=33		Severe RSV-LRTI N=16	
	n	% (95% CI)	n	% (95% CI)
Any[a]	13	39.4 (22.9–57.9)	6	37.5 (15.2–64.6)
Influenza A	0	0.0	0	0.0
Influenza B	0	0.0	0	0.0
Influenza A (H1)	0	0.0	0	0.0
Influenza A (H3)	0	0.0	0	0.0
Influenza A/California/7/2009	0	0.0	0	0.0
Human Adenovirus	0	0.0	0	0.0
Human metapneumovirus	0	0.0	0	0.0
Enterovirus	1	3.0	1	6.3
Human parainfluenza virus 1	0	0.0	0	0.0
Human parainfluenza virus 2	0	0.0	0	0.0
Human parainfluenza virus 3	0	0.0	0	0.0
Human parainfluenza virus 4	0	0.0	0	0.0
Human bocavirus	4	12.1	2	12.5
Rhinovirus	7	21.2	3	18.8
Human coronavirus OC43	1	3.0	0	0.0
Human coronavirus 229E	0	0.0	0	0.0
Human coronavirus NL63	0	0.0	0	0.0

RSV, respiratory syncytial virus; LRTI, lower respiratory tract illness based on the World Health Organization case definition; N, total number of nasal swab specimens from RSV-LRTI or severe RSV-LRTI cases; note, the number of swabs differs from the number of RSV-LRTI cases (32) as there was a case for which two swabs were taken (one initial swab + one after worsening); for severe RSV-LRTI (17 cases), the difference is due to one case starting as not severe and evolving to severe, with a swab taken before the episode evolved to severe; n/%,

number/percentage of nasal swab specimens from RSV-LRTI or severe RSV-LRTI cases positive for a given viral infection; CI, confidence interval.

[a]Any of the tested viruses (as listed in the table).

Supplementary Table 5. COVID-19 cases (suspected, probable, or confirmed) overall and by country

Assessment	Overall N=2181	Bangladesh N=195	Malaysia N=167	Philippines N=259	Thailand N=258	South Africa N=385	Argentina N=306	Brazil N=185	Colombia N=268	Mexico N=97	Panama N=61
Infants with ≥1 COVID-19 case	98 (4.5%)	1 (0.5%)	7 (4.2%)	10 (3.9%)	0 (0.0%)	2 (0.5%)	8 (2.6%)	39 (21.1%)	13 (4.9%)	3 (3.1%)	15 (24.6%)
Infants with multiple COVID-19 cases	15	0	2	1	0	0	0	6	2	0	4
Total number of COVID-19 cases	116	1	9	11	0	2	8	47	15	3	20

COVID-19 was diagnosed based on the World Health Organization definitions of 2021. N, total number of infants in the enrolled set.
